# Supplementary figures and images for: Discovery and functional interrogation of SARS-CoV-2 protein-RNA interactions
Source: Res Sq. 2022 Mar 17:rs.3.rs-1394331. Preprint. [Version 1] doi: 10.21203/rs.3.rs-1394331/v1 (PMC8936114; doi:10.21203/rs.3.rs-1394331/v1)

Supplementary Figure 1

a.

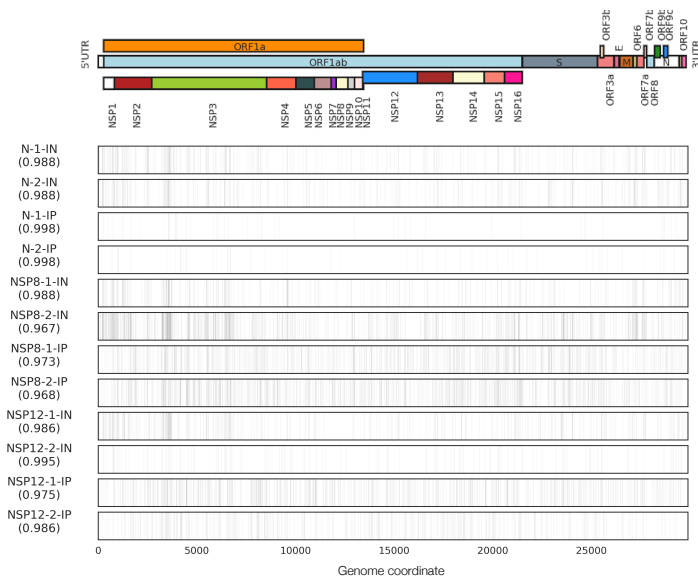

b.

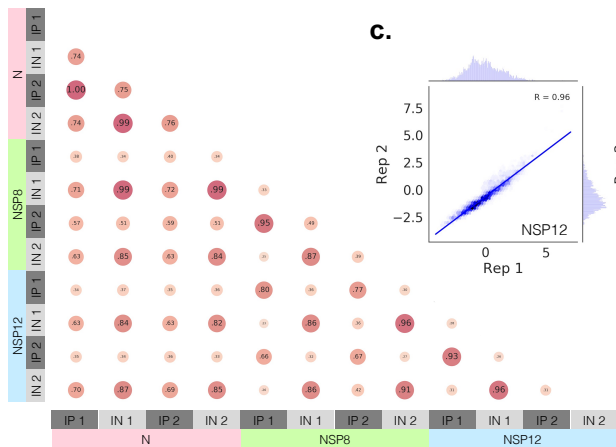

c.

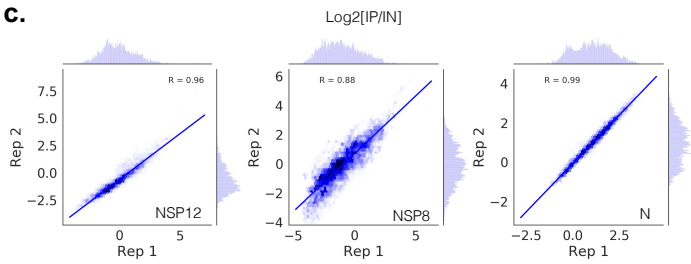

d.

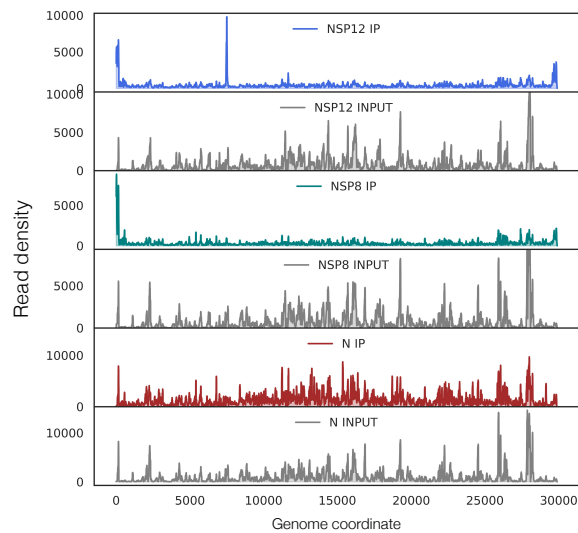

e.

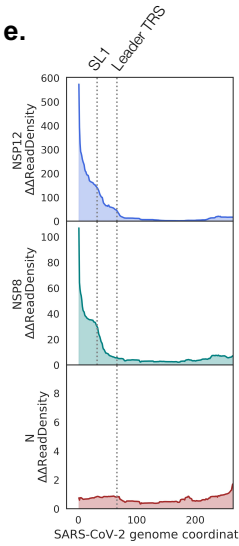

f.

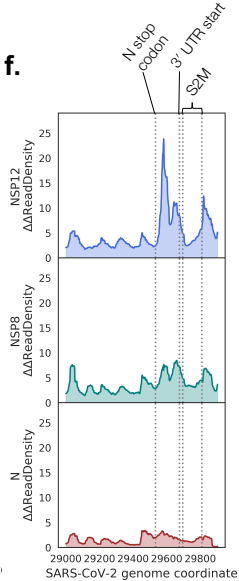

Supplement: Supplement 2 [file 8b0facb9d7bf34c88b87063c.pdf]

Supplementary Figure 2

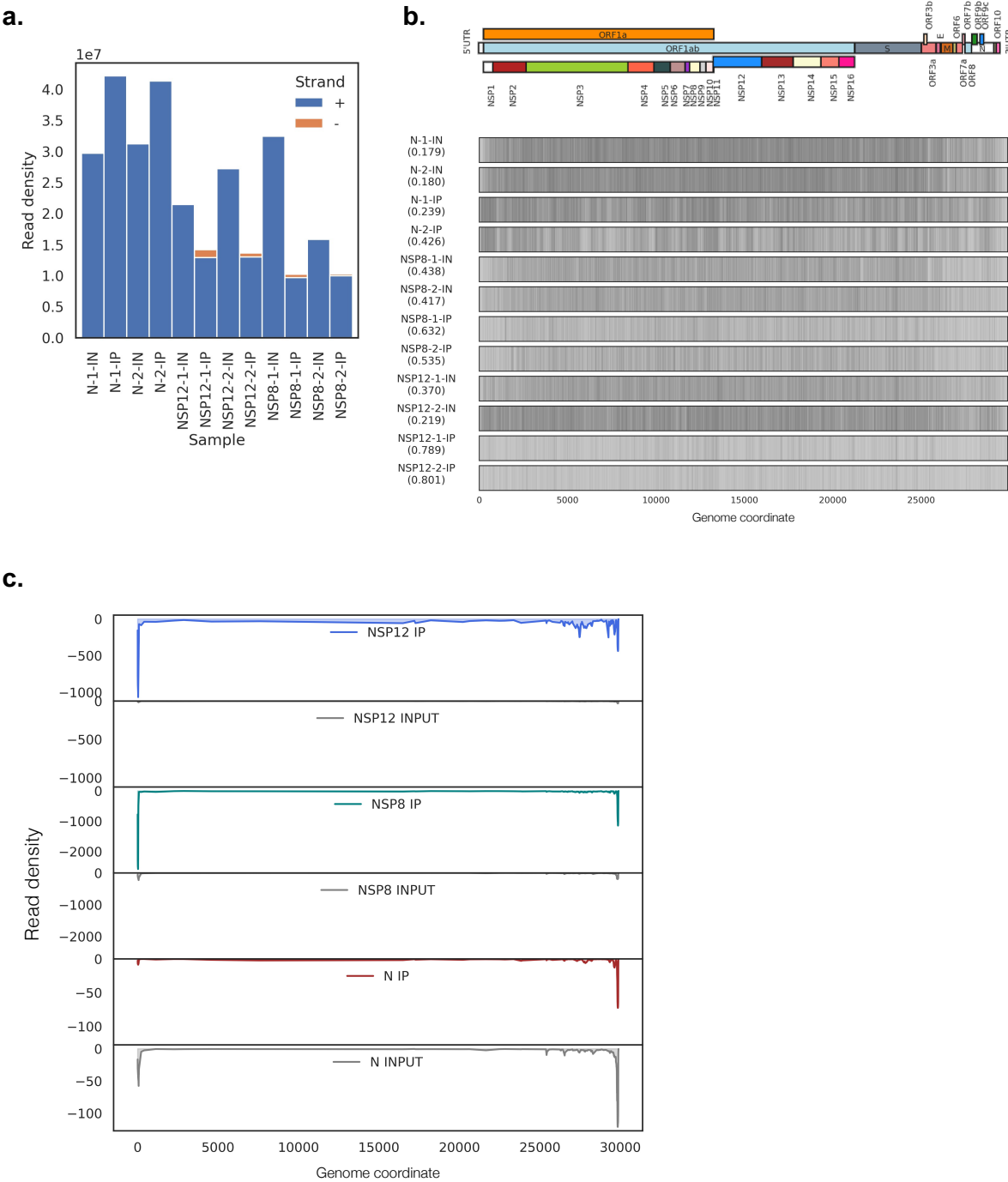

Supplement: Supplement 3 [file dc2b8eb2c887a650c4817c73.pdf]

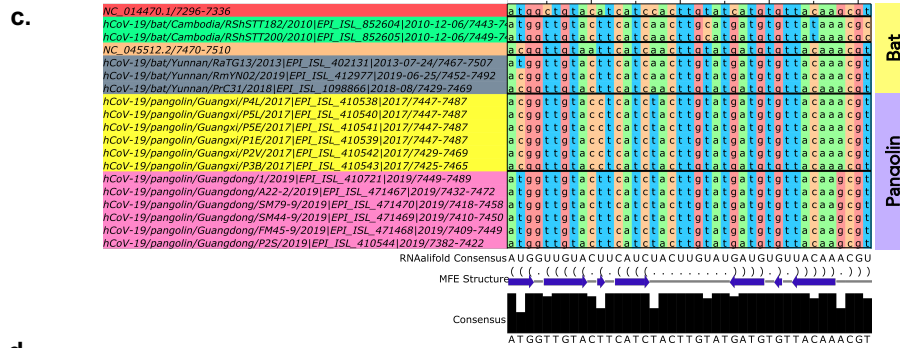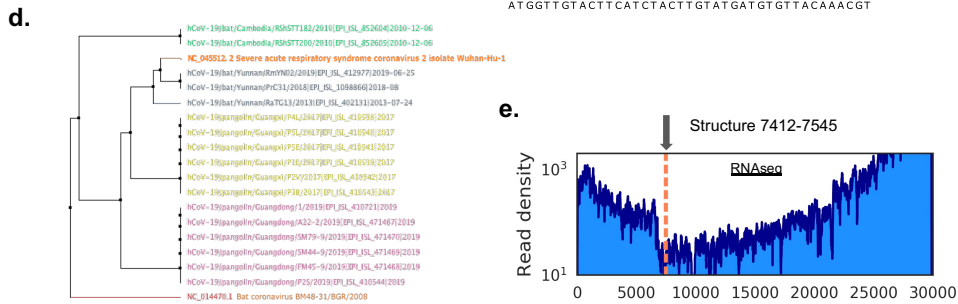

Supplement: Supplement 4 [file c66e1cca48c69687462cae0a.pdf]

Supplementary Figure 4

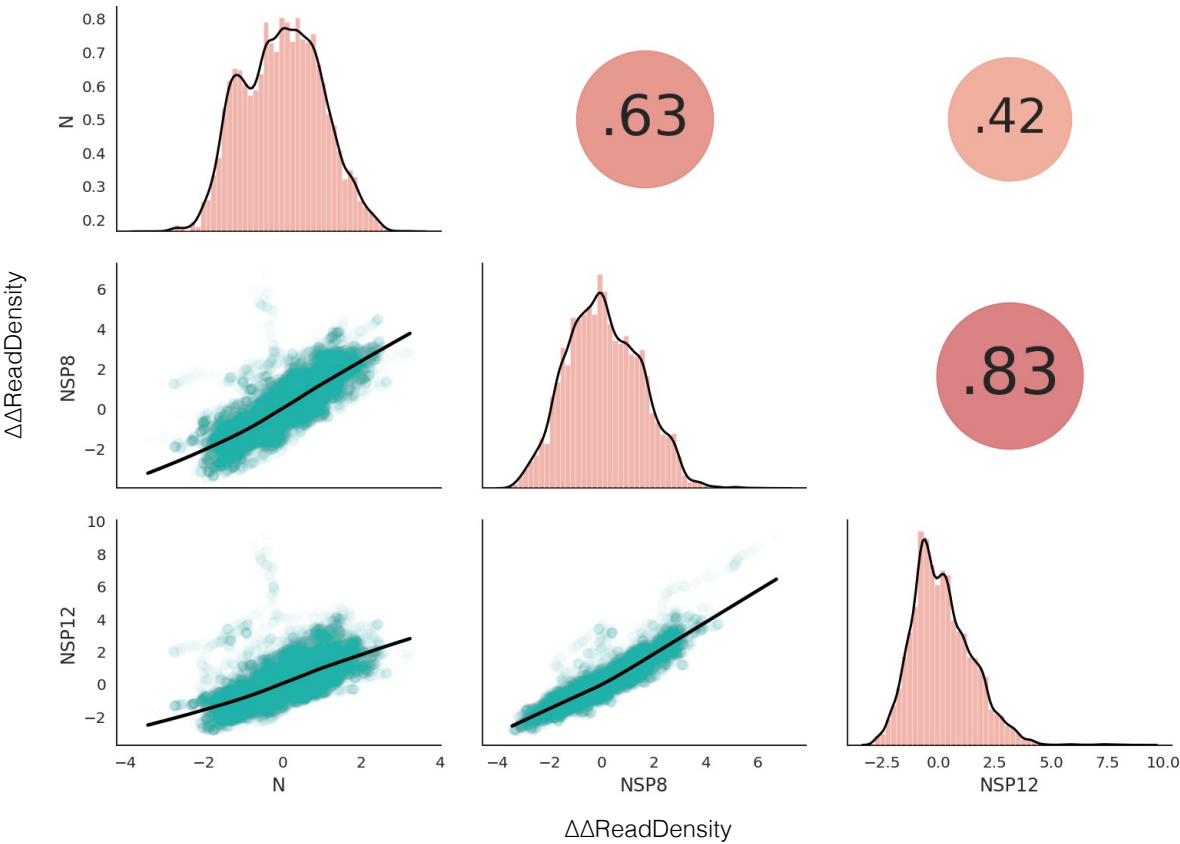

Supplement: Supplement 5 [file eefba804f0acb18da1decc49.pdf]

### Supplementary Figure 5

**a.**

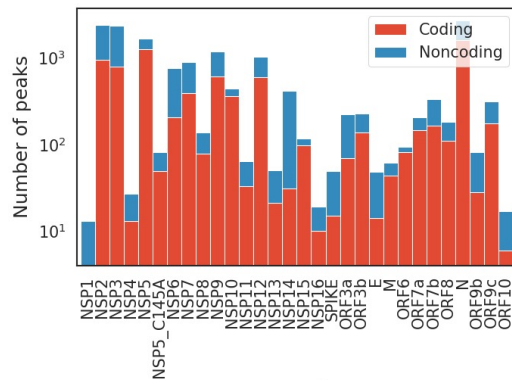

**b.**

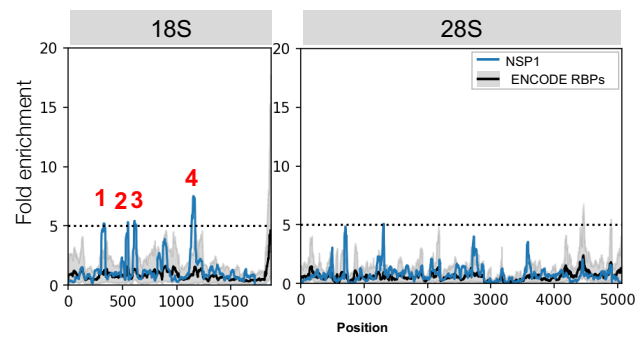

Supplement: Supplement 6 [file 2ff92031fd754a08ee4ee6db.pdf]

## Supplementary Figure 7

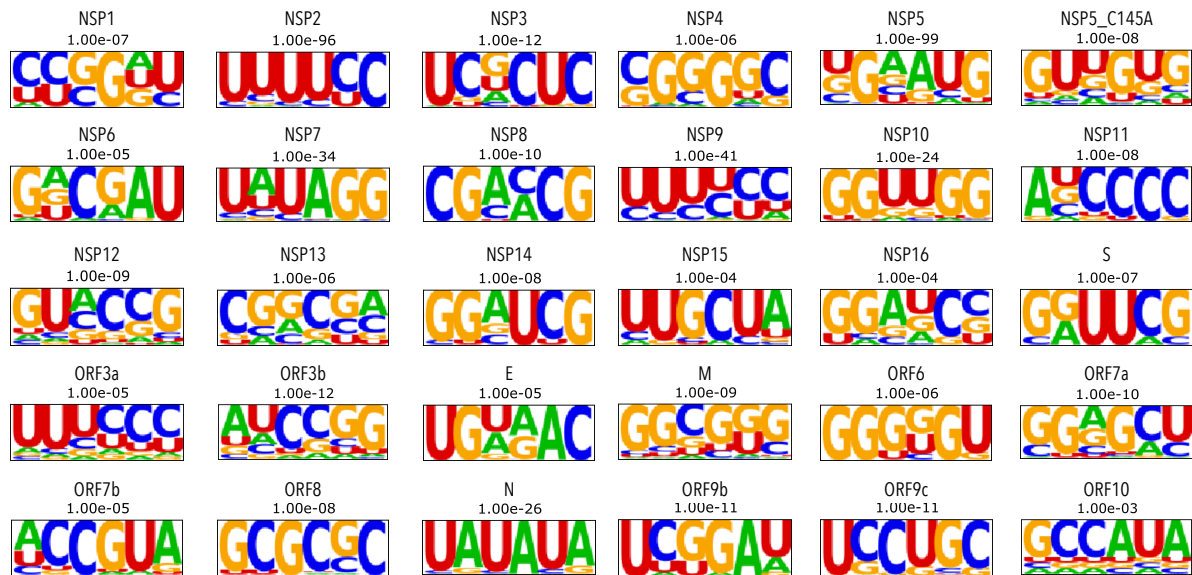

Supplement: Supplement 8 [file d08ad3dcddf2bb668dabfbea.pdf]

Supplementary Figure 8

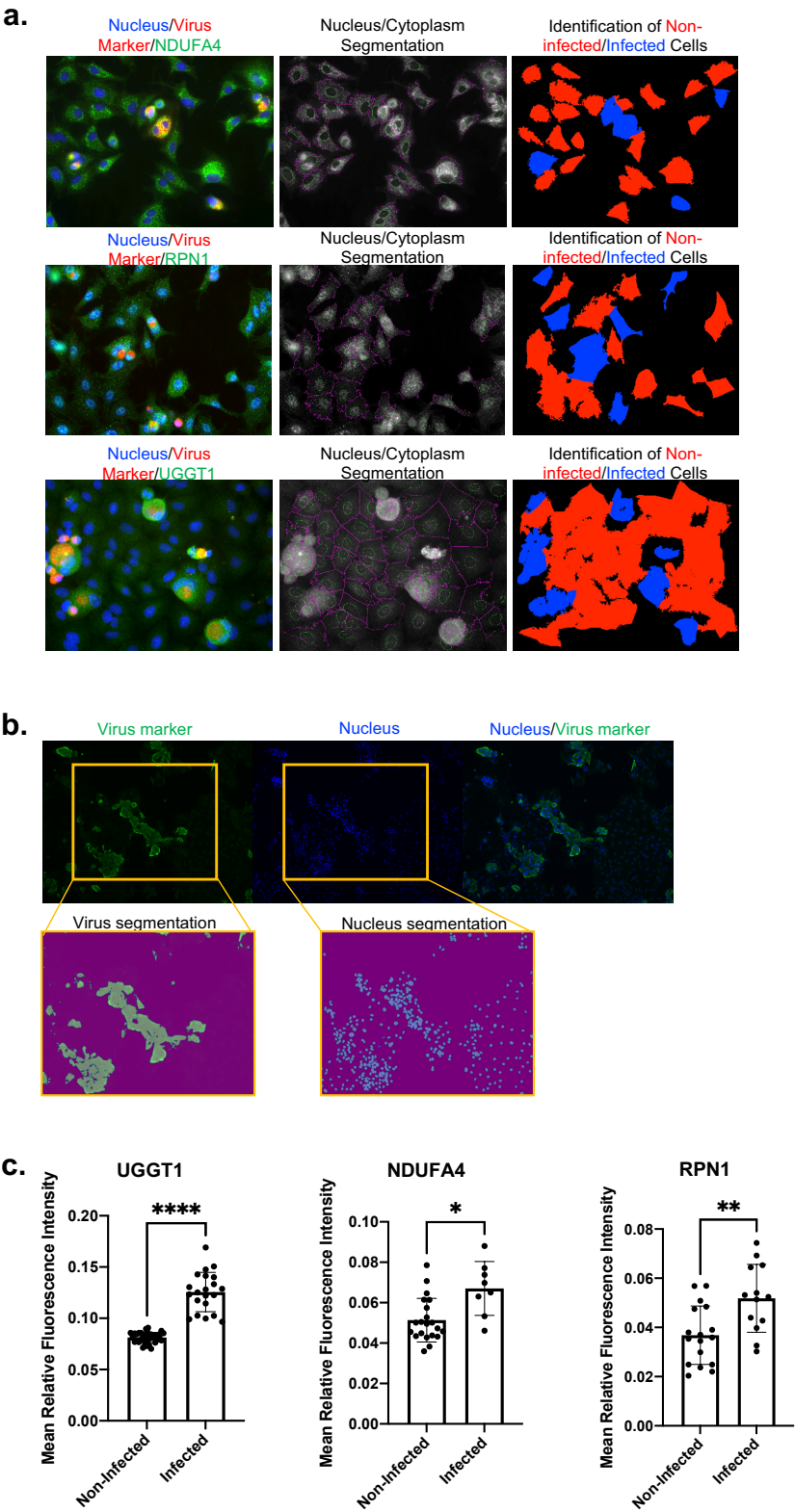

Supplement: Supplement 9 [file c394a768e25b146167509356.pdf]

Supplementary Figure 9

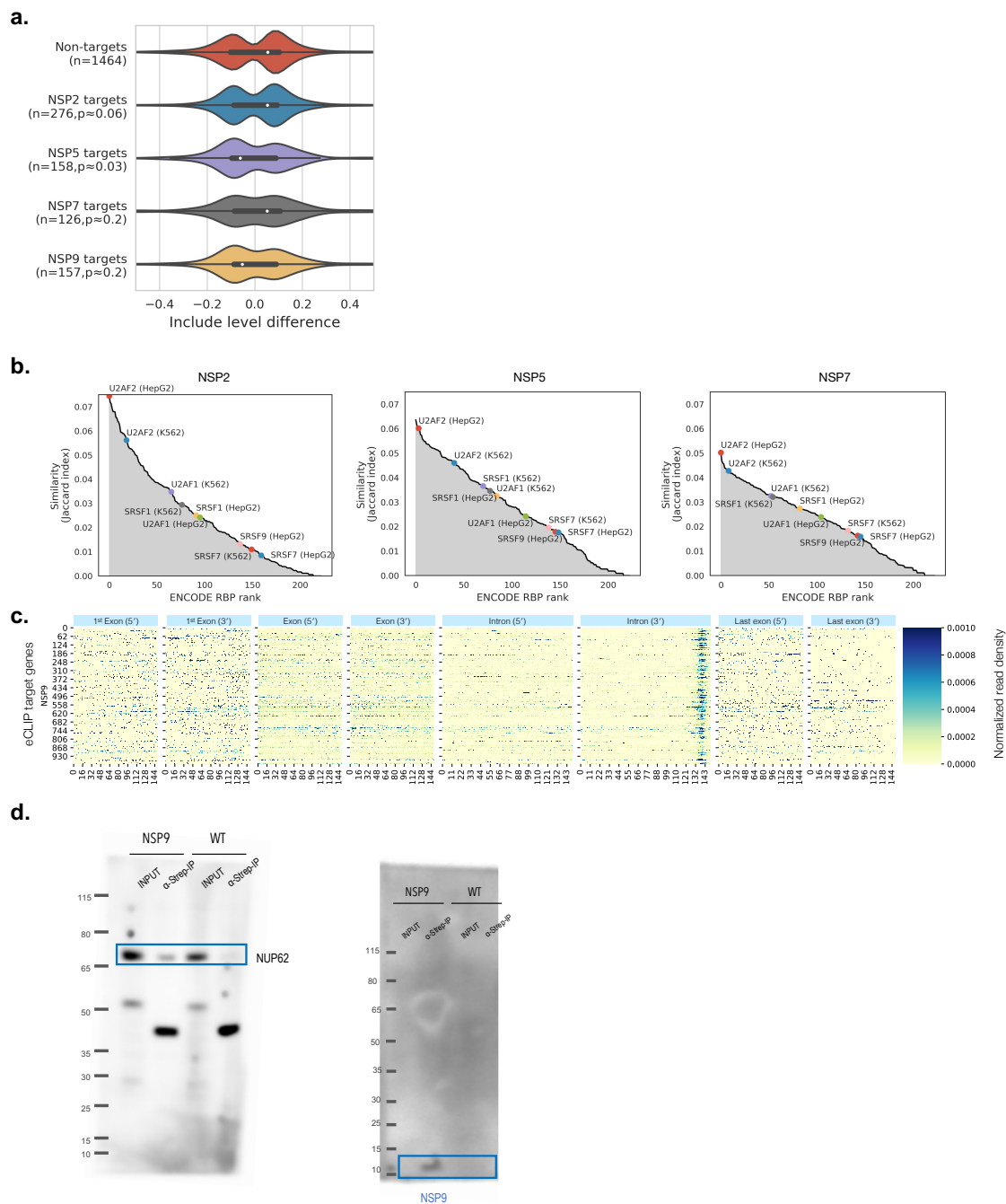

Supplement: Supplement 10 [file abf6a64edcf1a7895ae04491.pdf]
