# Supplementary material for: Discovery and functional interrogation of SARS-CoV-2 protein-RNA interactions
Source: Res Sq. 2022 Mar 17:rs.3.rs-1394331. Preprint. [Version 1] doi: 10.21203/rs.3.rs-1394331/v1 (PMC8936114; doi:10.21203/rs.3.rs-1394331/v1)

Supplementary Figure 6

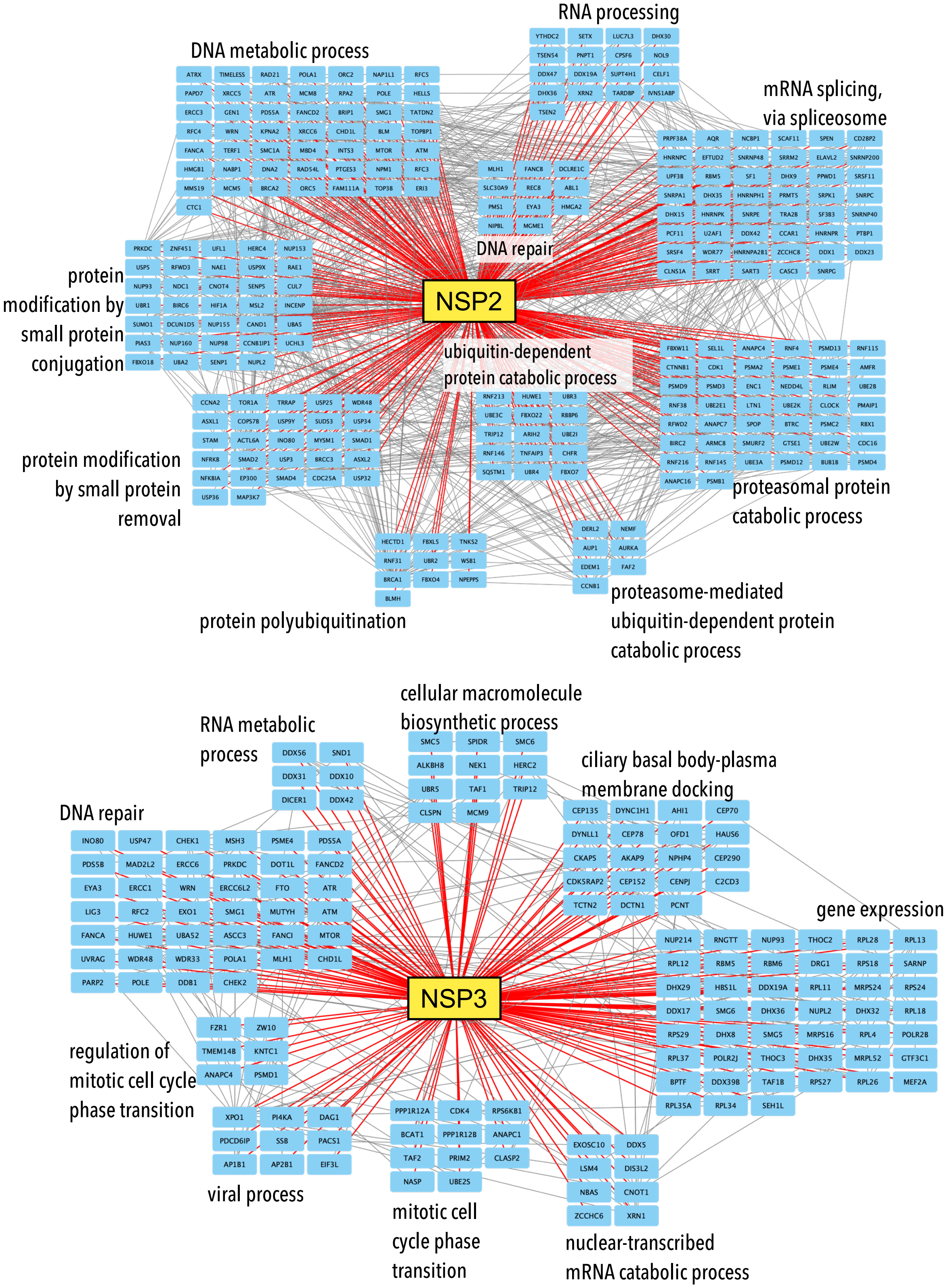

Supplementary Figure 6

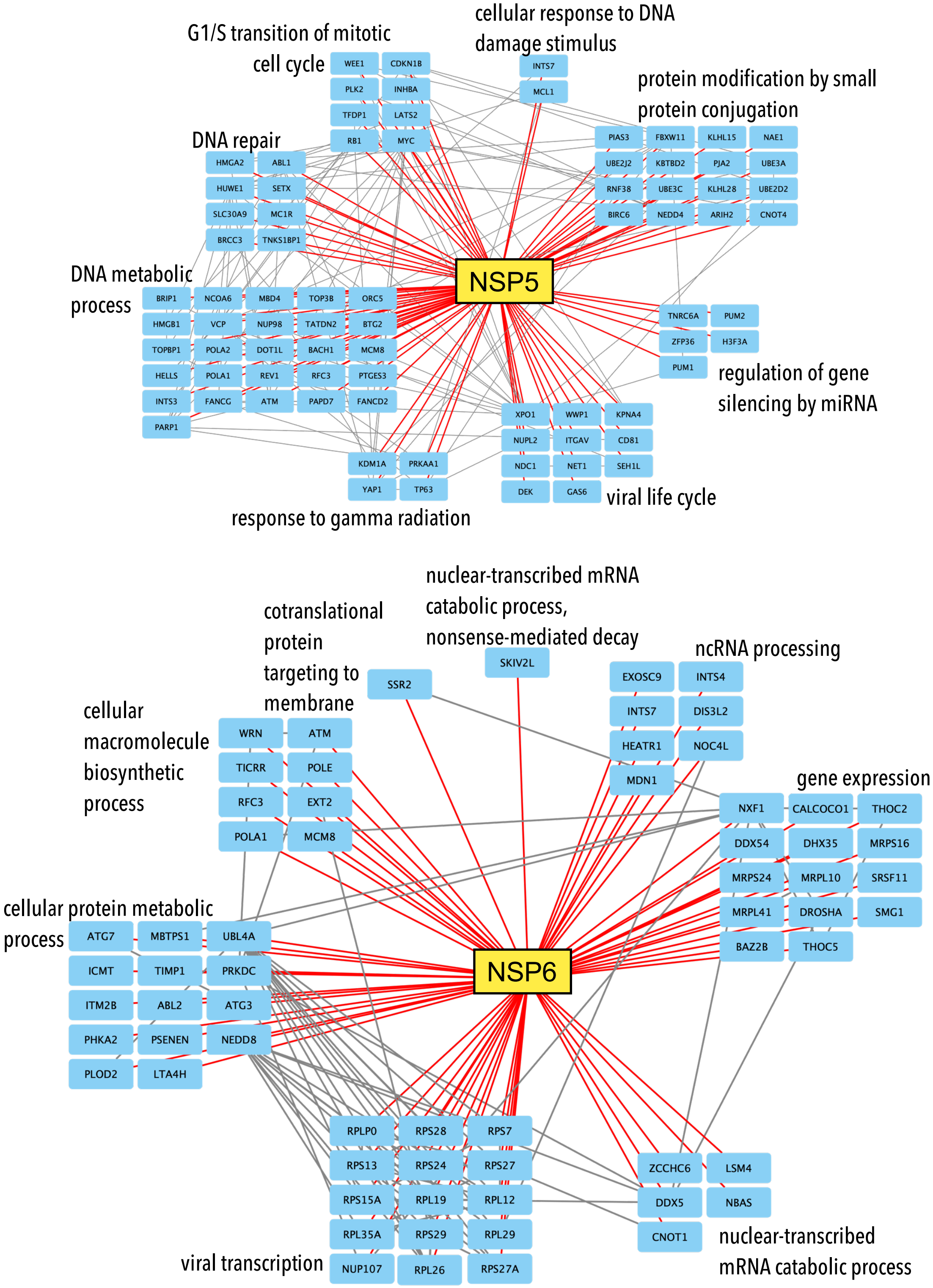

Supplementary Figure 6

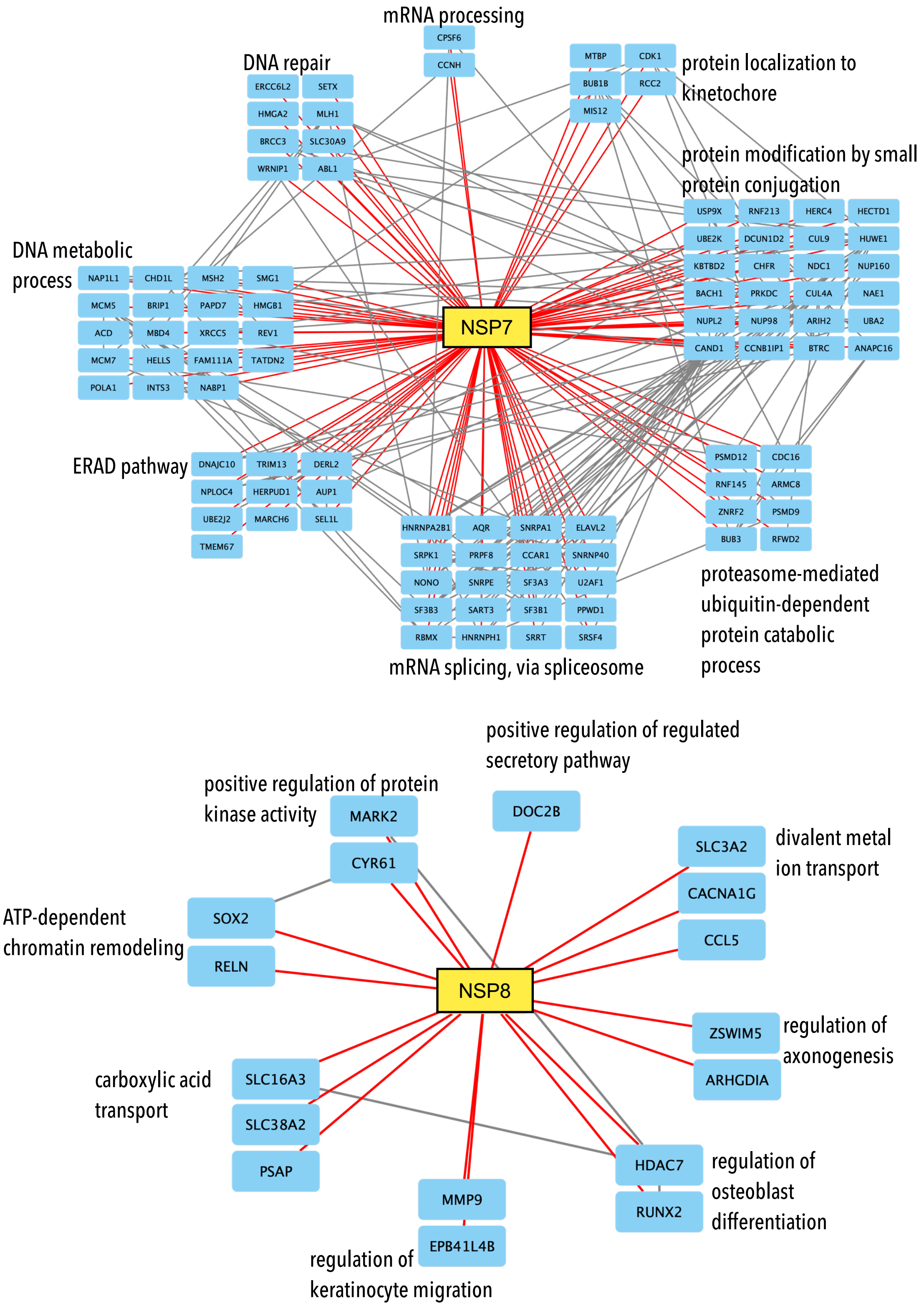

Supplementary Figure 6

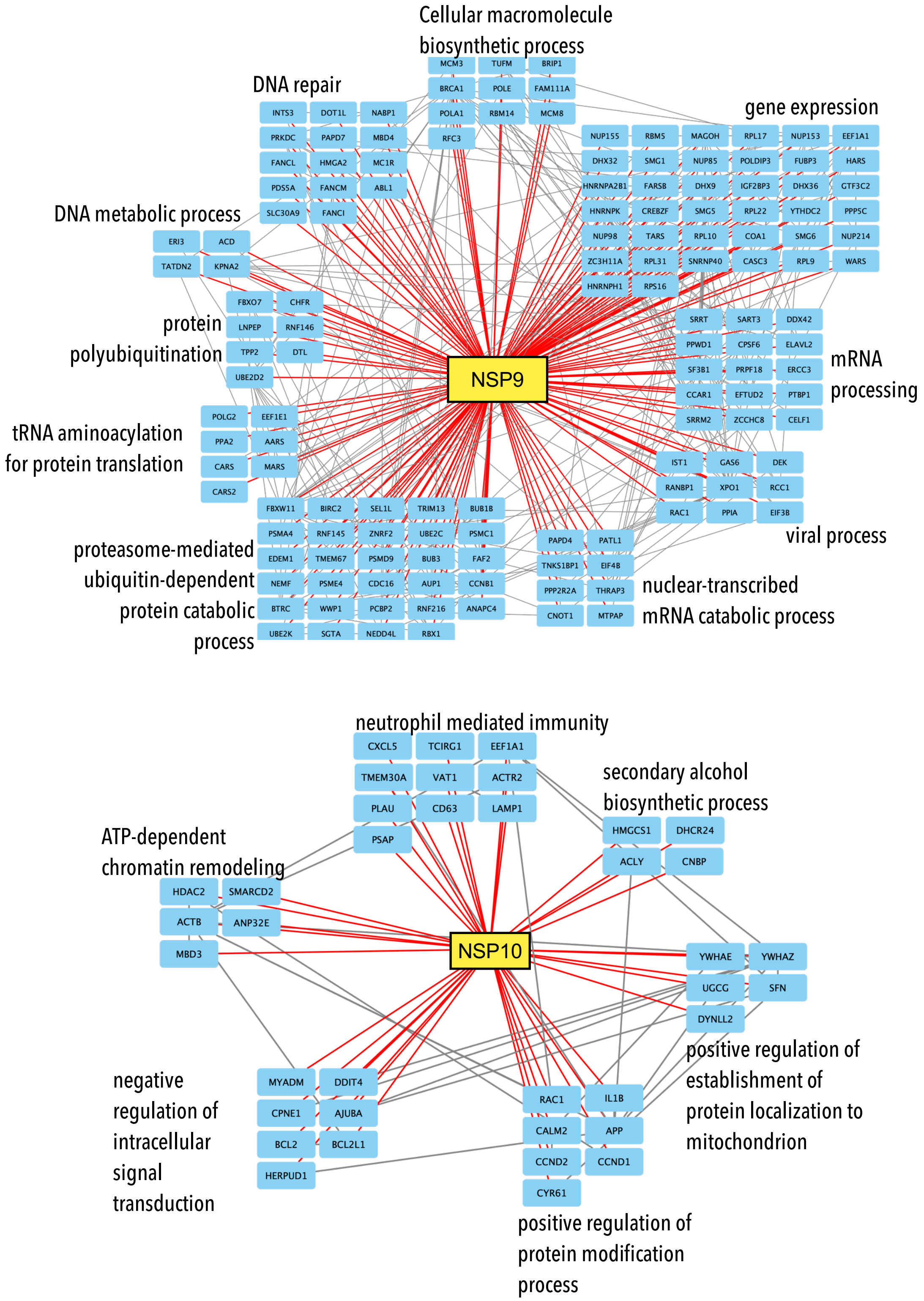

Supplementary Figure 6

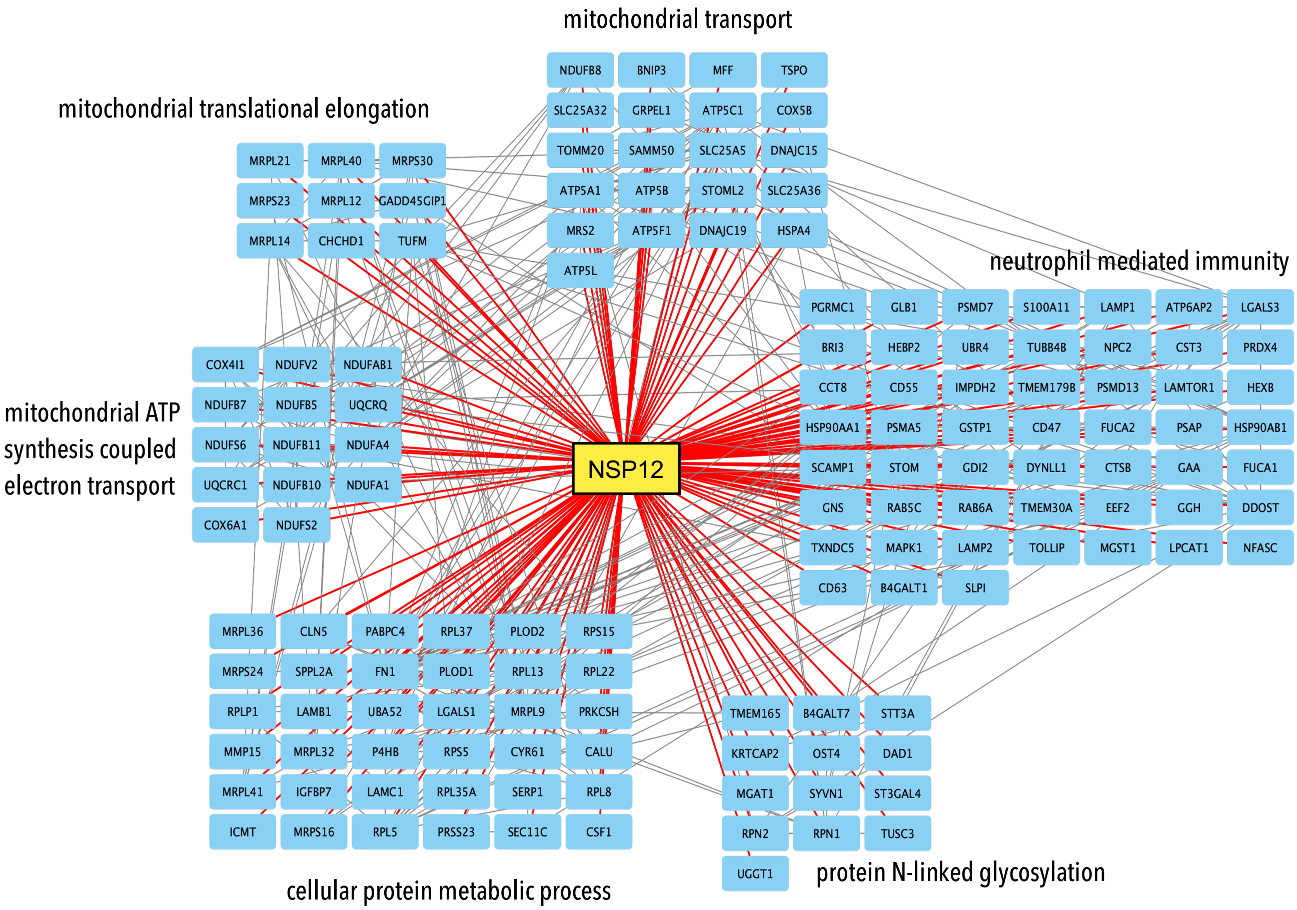

Supplementary Figure 6

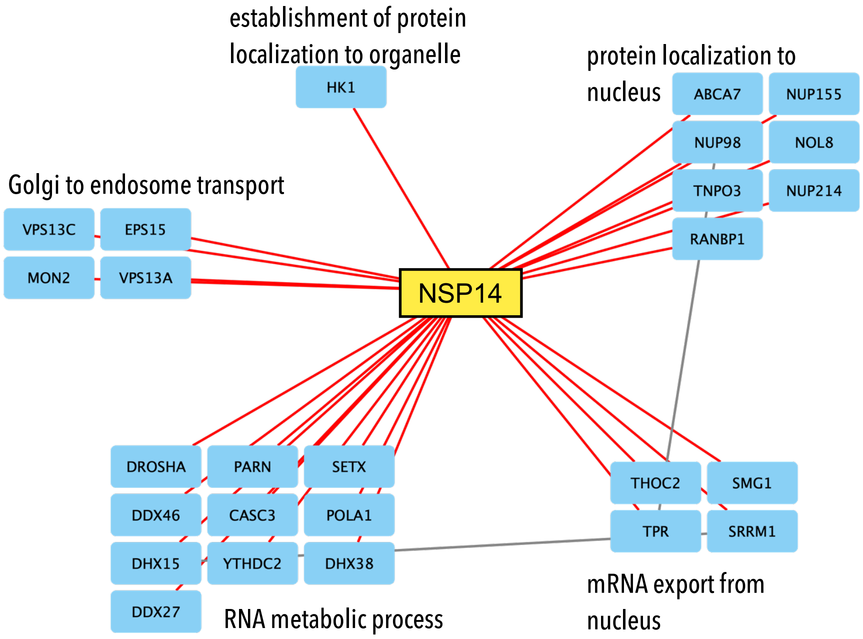

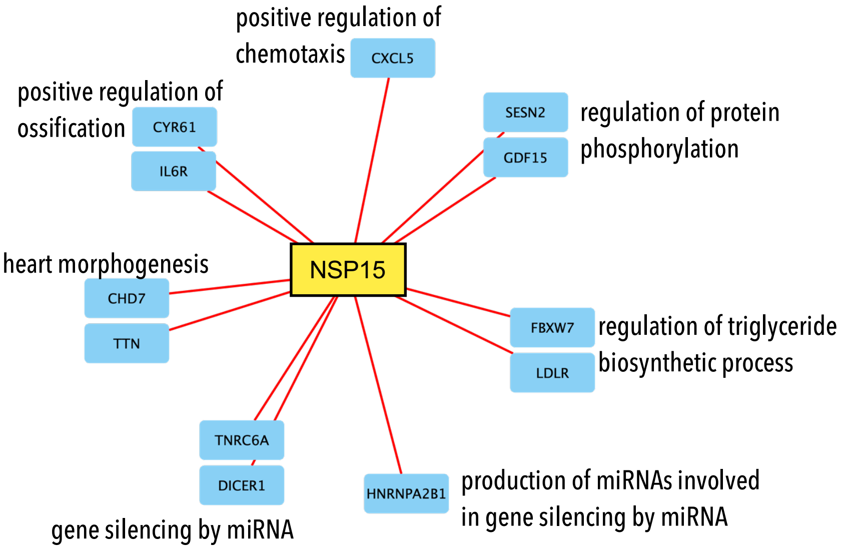

Supplementary Figure 6

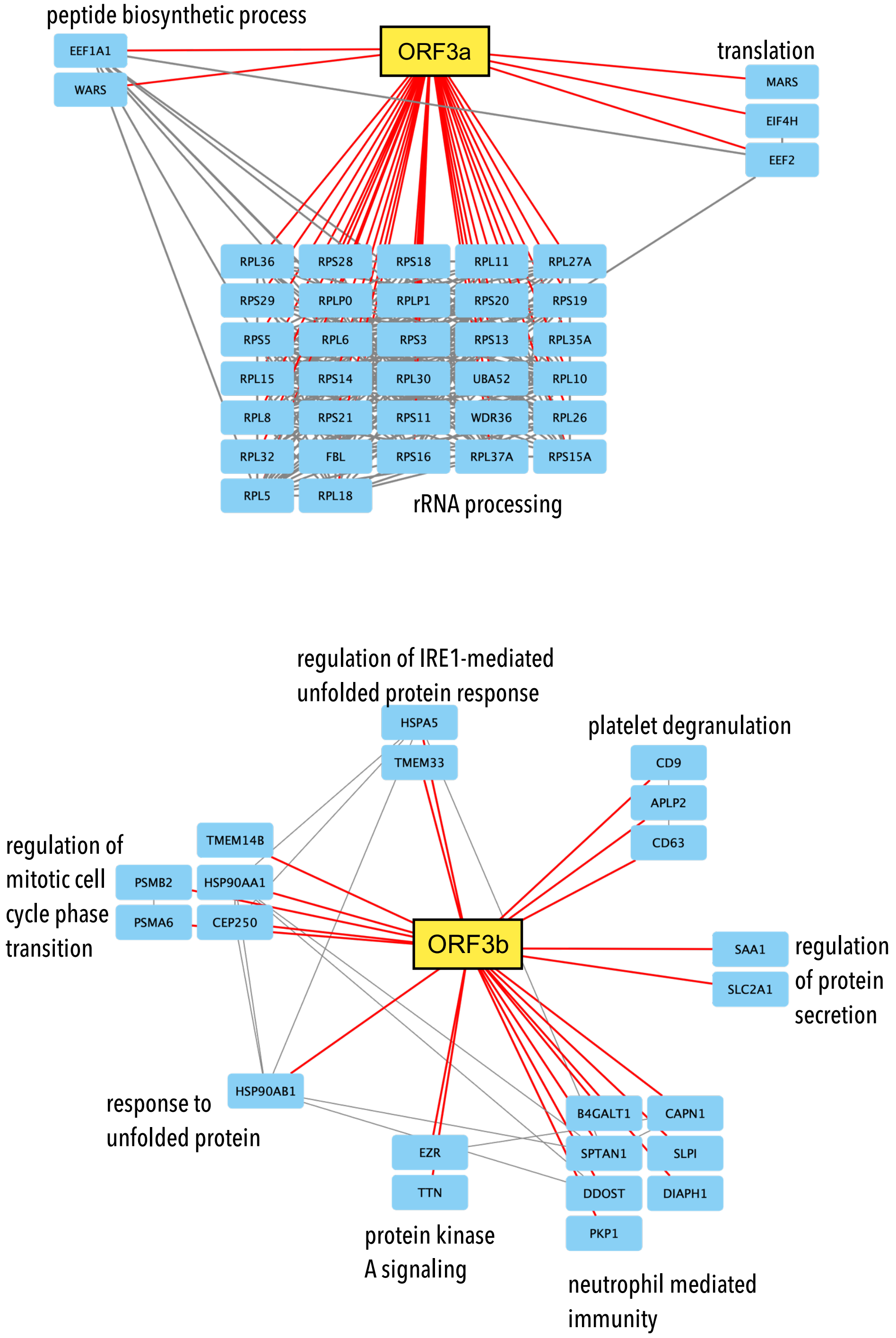

Supplementary Figure 6

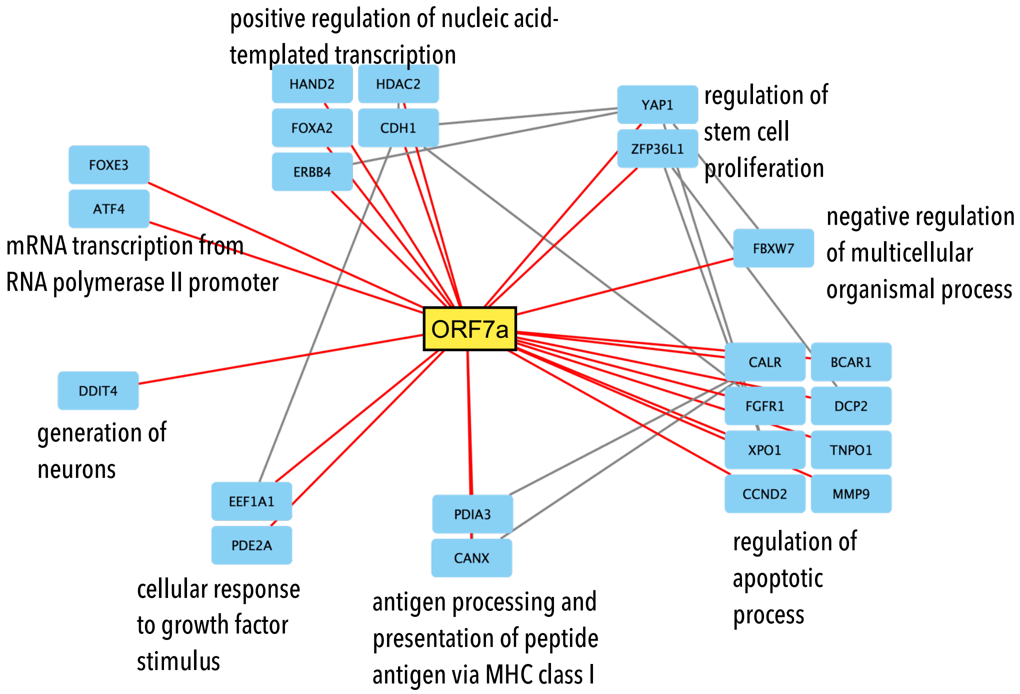

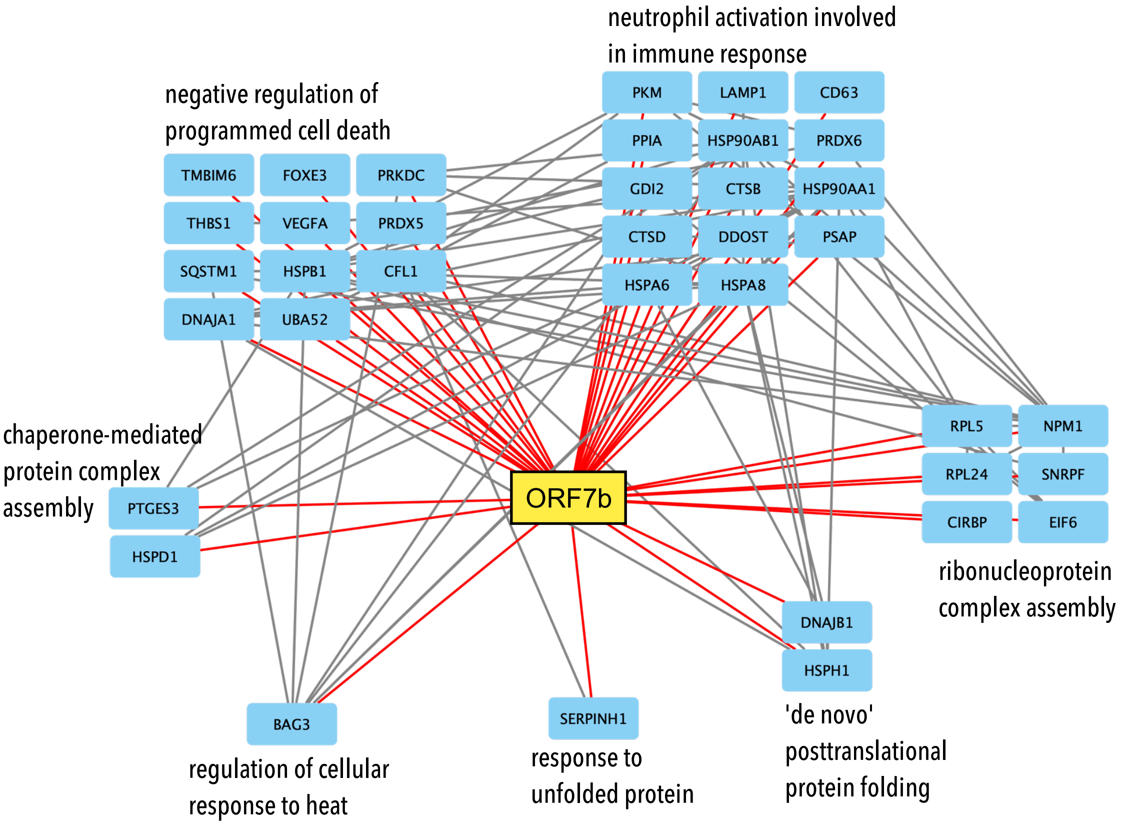

Supplementary Figure 6

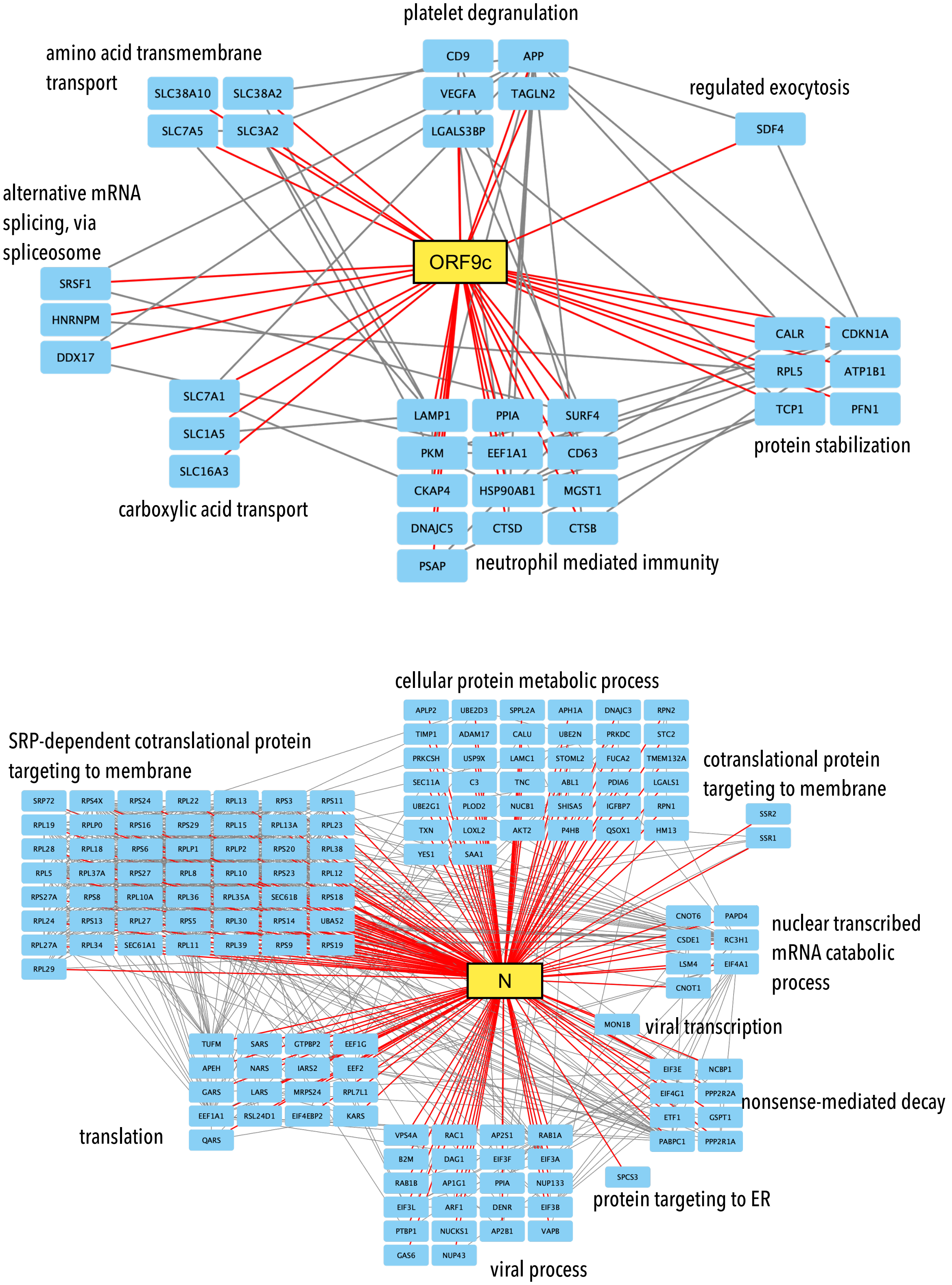

Supplement: Supplement 7 [file ef1ffcd95cab4a5b42e44785.pdf]
